# Dendrobine ameliorates mitophagy-mediated endothelial senescence in diabetic kidney disease through activating the SIRT1/FOXO3a pathway

**DOI:** 10.1186/s13020-025-01234-7

**Published:** 2025-10-18

**Authors:** Yan Zhang, Xuan Qi, Rui-Xuan Sun, Yong-Fang Gong, Hong-Kai Yang, Wu-Ling Wang, Yan Yang, Yong-Sheng He, Yu-Sheng Shi

**Affiliations:** 1 Ma’anshan People’s Hospital, Ma’anshan, 243000 China; 2 Department of Pharmacology, School of Basic Medicine, Anhui Medical University, Hefei, 230032 China; 3 College of Health-Preservation and Wellness, Dalian Medical University, Dalian, 116044 China; 4 Department of Nuclear Medicine, the Second Affiliated Hospital of Dalian Medical University, Dalian, 116011 China; 5 Department of Institute (College) of Integrated Medicine, Dalian Medical University, Dalian, 116044 China

**Keywords:** Diabetic kidney disease, Dendrobine, SIRT1/FOXO3a pathway, Senescence

## Abstract

**Background:**

Glomerular senescence plays a vital role in the pathogenesis of diabetic kidney disease (DKD). Dendrobine (Den) exhibits anti-senescence properties and nephroprotective effects; however, its precise impact and underlying mechanisms in ameliorating glomerular senescence in DKD remain unclear.

**Methods:**

The db/db mice were orally administered Den (10 and 30 mg/kg) for 8 weeks to evaluate its nephroprotective effect. Network pharmacology analysis was performed to investigate its underlying mechanisms. Senescence-associated β-galactosidase (SA-β-Gal) staining, Western blot, and RT-qPCR were employed to evaluate the beneficial effects of Den on inhibiting senescence in both in vivo and in vitro settings. The effects of Den on mitophagy were evaluated using transmission electron microscopy (TEM) and Western blot. RNA-seq was conducted to explore the molecular mechanisms underlying Den’s amelioration of mitophagy-mediated endothelial senescence. siRNA and a pharmacological inhibitor of SIRT1 were utilized to validate the role of the SIRT1/FOXO3a pathway in this process.

**Results:**

Biochemical and histological analyses indicated Den protected against renal injury in DKD mice. Network pharmacology analysis suggested Den’s beneficial nephroprotective effects were associated with cellular senescence. SA-β-Gal staining showed that Den reduced the positive area of SA-β-Gal. Western blot and RT-qPCR results revealed that Den decreased the levels of senescence-associated proteins (p21 and p16) as well as secretory phenotype markers (IL-6, IL-8, IL-1β, MMP1, MMP3, and MMP10). Additionally, Den improved mitophagy levels, as evidenced by increased mitochondrial mean length and mitophagy-associated proteins (PINK1, Parkin, NIX, and BNIP3), along with a decreased proportion of damaged mitochondria. RNA-seq analysis indicated *SIRT1* mRNA levels were significantly upregulated following Den treatment. Furthermore, siRNA-mediated knockdown of SIRT1 and Selisistat administration demonstrated that Den’s inhibition of mitophagy-mediated endothelial senescence was associated with the activation of the SIRT1/FOXO3a pathway.

**Conclusions:**

These findings demonstrate that Den ameliorates endothelial senescence in DKD by enhancing mitophagy through activating the SIRT1/FOXO3a pathway, highlighting a promising therapeutic strategy for DKD patient.

## Introduction

Diabetic kidney disease (DKD), characterized by the presence of albuminuria and a decreased estimated glomerular filtration rate (eGFR), represents a severe microvascular complication associated with diabetes [1, 2]. Glomerulosclerosis, basement membrane thickening, and tubular injury are the primary pathological features observed in DKD. Epidemiological data indicate that approximately 40% of patients with diabetes progress to DKD [3], which is a prominent cause of chronic kidney disease (CKD) and contributes significantly to elevated morbidity and mortality [4]. With the rising global prevalence of diabetes and extended life expectancy [5], there is an urgent need for effective and practical intervention strategies to prevent the prevalence of DKD. However, clinical trial evidence suggests that optimizing glycemic control, as recommended for diabetic patients, provides only limited renoprotection benefits [6]. Hence, the discovery of safe and efficacious medications targeting novel pathologic mechanisms is essential for the optimal management of patients with DKD.

Cellular senescence is an irreversible state of cell cycle arrest, characterized by enhanced activity of senescence-associated β-galactosidase (SA-β-Gal), secretion of senescence-associated secretory phenotype (SASP), and activation of the DNA damage response [7, 8]. Accumulating evidence indicates that disturbed glucose metabolism under diabetic conditions accelerates senescence in multiple tissues, contributing to the development of diabetic complications [7, 9, 10]. Clinical findings have confirmed elevated cellular senescence in DKD, as evidenced by increased SA-β-Gal-positive staining and p16 expression in type 2 diabetic nephropathy biopsies compared to age-matched controls [11]. Notably, glomerular p16 expression correlates with proteinuria, whereas tubular p16 expression is associated with key risk factors for diabetes [11]. Several studies have demonstrated that targeting renal tubular p21 expression reverses renal hyperglycemic memory-induced senescence in patients with DKD [12]. However, the effects of alleviating glomerular senescence on slowing DKD progression remain unclear. Therefore, it is necessary to elucidate the potential pathogenic mechanisms underlying glomerular senescence and guide the research and discovery of novel drugs for DKD patients.

Mitophagy, encompassing the irreversible fission of damaged mitochondrial fragments, formation of mitophagosomes, fusion and degradation of mitophagosomes within lysosomes, is a physiological process that maintains cellular homeostasis by selectively eliminating impaired mitochondria [13, 14]. Under DKD conditions, clinical evidence has demonstrated decreased levels of mitophagy, which compromises the efficiency of oxidative phosphorylation and increases reactive oxygen species (ROS) production, thereby promoting cellular senescence [15]. Numerous studies have highlighted the pivotal role of deacetylases in regulating autophagic flux [16–18]. For instance, placental mesenchymal stem cells attenuate podocyte injury in DKD by modulating mitophagy through the SIRT1 pathway [19]. Glomerular endothelial cells, which are rich in mitochondria, exhibit mitochondrial dysfunction-mediated senescence. However, there remains a paucity of therapeutic agents targeting endothelial cells to enhance mitophagy for ameliorating glomerular senescence.

The traditional Chinese medicine (TCM) *Dendrobium officinale*, renowned for its life-prolonging properties, is extensively utilized in both daily healthy maintenance and clinical practice to prevent diabetes [20]. Previous studies have demonstrated that *D. officinale* effectively mitigates renal inflammation, improves insulin resistance, and inhibits renal fibrosis, thereby slowing the progression of DKD [21, 22]. Dendrobine (Den), a pyrrolizidine derivative alkaloid isolated from *D. officinale*, exhibits hypoglycemic and anti-senescence effects [20]. A recent study has revealed that Den modulates STAT3/FOXO signaling pathway to attenuate mitophagy and senescence in endothelial cells induced by oxidized low-density lipoprotein [23]. Moreover, Den has been reported to upregulate the expression of SIRT1 [24]. Based on these findings, we hypothesized that Den ameliorates mitophagy-mediated endothelial senescence in DKD through activating the SIRT signaling pathway. In this study, SA-β-Gal staining was performed along with assessment of SASP levels and expression of senescence-associated proteins to evaluate the impact of Den on senescence in DKD. Knockout models and a pharmacological inhibitor targeting SIRT1 were used to investigate the underlying mechanism responsible for Den’s beneficial effect on endothelial senescence. These results will elucidate the advantages associated with preventing glomerular senescence while also providing a potential pharmacologic intervention strategy for DKD patients.

## Materials and methods

### Animals

Male db/db mice (12 weeks old) and age-matched db/m mice were purchased from Gempharmatech (Jiangsu, China). All animal procedures were approved by the Ethics Review Committee for Animal Experimentation of Ma’anshan People’s Hospital (V1.0 20,211,109). Mice were housed under a conditional environment with a temperature of 25 ± 2 ℃, humidity of 60% ± 5%, and a 12 h light/dark cycle. Following a one-week acclimatization period, db/db mice were divided into four groups as follows (*n* = 8 per group): db/db group, Den-L group (10 mg/kg/d); Den-H group (30 mg/kg/d), and Dapa group (2 mg/kg/d), with age-matched controls using db/m mice. For validation experiments, db/db mice were divided into three groups as follows (*n* = 6 per group): db/db group, Den group (30 mg/kg/d), Den + Selisistat group (Den: 30 mg/kg/d; Selisistat: 10 mg/kg/d, i.p.), with age-matched control using db/m mice. Serum, urine, and kidney tissues were collected from all mice after 8 weeks of drug treatment.

### Biochemical testing

The serum glucose, creatinine, urea nitrogen, and 24-h urine albumin levels were evaluated by commercial kits following the manufacturer’s description (Nanjing Jiancheng Bioengineering Research Institute Co., Ltd, Nanjing, China).

### Pathological analysis

Mouse kidney tissues were fixed with 4% paraformaldehyde (PFA), embedded in paraffin, cut into Sections (5 μm), and stained with hematoxylin–eosin (H&E), Periodic Acid-Schiff (PAS), and Masson solution (Solarbio, Beijing, China) according to a previous study.

### Network pharmacology analysis

GeneCards (https://www.genecards.org) and OMIM (https://www.omim.org) databases were utilized to identify potential targets associated with the keywords “diabetic kidney disease”. Subsequently, Swiss Target Prediction (http://swisstargetprediction.ch) and Pharmmapper (https://www.lilab-ecust.cn/pharmmapper) databases were employed to predict possible therapeutic targets for Den. The intersecting genes were further queried in the STRING (https://cn.string-db.org/) database, and the resultant gene interaction network was visualized and analyzed using Cytoscape (3.7.1) software. The top 20 targets with the highest degree value are considered core targets. Finally, the core targets were imported into the Metascape (https://metascape.org) and DAVID (https://david.ncifcrf.gov) databases for comprehensive enrichment analysis.

### Senescence-associated β-galactosidase staining

Mouse kidney tissues were frozen with liquid nitrogen, embedded in optimal cutting temperature compound (OST, Sakura, USA), and cut into Sections (5 μm). The sections and cultured cells were stained with SA-β-Gal solution (Solarbio, Beijing, China) according to the manufacturer’s description.

### Cell culture and treatment

The human umbilical vein endothelial cells (HUVECs, ATCC) were cultured in DMEM/F12 basic medium (KeyGEN, Nanjing, China) supplemented with 10% fetal bovine serum (Gibco, USA) at 37℃ and 5% CO_2_. HUVECs were induced with High-glucose (HG, 40 mmol/L) for 48 h to establish a cell model in vitro, and cultured with 40 mmol/L mannitol were used as iso-osmotic non-glucose control.

### Cell viability

HUVECs were plated into 96-well plates (NEST, Wuxi, China) and treated with Den (0, 5, 10, 20, 40 μM) for 24 h. Cell counting kit-8 solution (10 μL, KeyGEN, Nanjing, China) was added to each well. Two hours later, the optical density at 450 nm was measured using a microplate reader (Mindray, Shenzhen, China).

### Transmission electron microscopy (TEM)

Cultured cells were collected and fixed with 2.5% glutaraldehyde, followed by infiltration with 1% osmium tetroxide in cacodylate buffer. Subsequently, the samples were dehydrated and embedded in epoxy resin. For visualization, the sections were stained with a solution containing 3% uranyl acetate and lead citrate, and images were acquired using a JEM-1400 TEM.

### RNA sequencing

Total RNA was extracted separately from HG-induced HUVECs with or without Den treatment using the RNA isolater Total RNA Extraction Reagent (Vazyme, Nanjing, China), separately. raw sequencing data were processed using R (v 4.2.0). Sequence quality was assessed, and low-quality reads were filtered using FastQC (v 0.12.1). Samples failing quality thresholds (> 10% bases with Phred score < 20 or > 5% adapter contamination) were excluded from downstream analysis. Reads were then aligned to the reference genome using HISAT2 (v 1.24.0). Transcript assembly and quantification of gene-level read counts were performed using Stringtie (v 2.2.3). Technical reproducibility was rigorously assessed through inter-replicate concordance quantified using Pearson correlation coefficients, principal component analysis (PCA) of variance-stabilized counts, and hierarchical clustering with Euclidean distance metrics to identify outlier samples and batch effects. Data normalization was implemented in an analytical cascade: compositional bias was corrected using the TMM method with edgeR (v4.6.3) to adjust for library size differences, and variance stabilization was achieved through regularized logarithm transformation with DESeq2 (v1.46.1) to ensure homoscedasticity for downstream analyses. EdgeR algorithm was applied to filter differentially expressed genes (DEGs) through logFC > 1 or < − 1, and P value < 0.05. Cluster analysis and principal component analysis (PCA) were performed based on DEGs. Gene Ontology (GO) enrichment analyses were performed using the DAVID database. The foldchange of the TPM was analyzed by gene set enrichment analysis (GSEA) with GO dataset.

### Western blot

Proteins were extracted from kidney tissue and cells using RIPA lysis buffer (Beyotime, Shanghai, China) supplemented with PMSF (Beyotime, Shanghai, China) on ice, followed by centrifugation at 12,000*g* for 10 min at 4 ℃. Protein content was determined using the Enhanced BCA Protein Assay Kit (Beyotime, Shanghai, China) according to the manufacturer’s instructions. Equal protein samples were subjected to SDS-PAGE, transferred onto nitrocellulose (NC) membrane, blocked with 5% BSA (Bioss, Beijing, China) for 2 h, and incubated with primary antibodies as follow at 4 °C overnight: p16, p21, PINK1, Parkin, NIX, BNIP3, SIRT1, FOXO3a, and ac-FOXO3a, respectively. Next, the membranes were incubated with a horseradish peroxidase (HRP)-conjugated secondary antibody (Wanlei, Shenyang, China) and visualized using a commercial enhanced chemiluminescence kit (Wanlei, Shenyang, China). Grey values were quantified using Image J software and normalized to expressions of β-actin as an internal standard.

### RT-qPCR assay

Total RNA from cell and kidney samples was isolated using RNA isolator Total RNA Extraction Reagent (Vazyme, Nanjing, China) and reverse-transcribed by All-in-One First-Strand Synthesis Master Mix (Best Enzymes, Lianyugang, China). Taq-HS SYBR Green qPCR Premix (Best Enzymes, Lianyugang, China) was applied to transcribe the cDNA in a Real-Time PCR system.

### Statistical analysis

All data were expressed as Mean ± SEM, and multiple groups were compared using One-way ANOVA. The statistical plots were drawn by GraphPad Prism 9 software. *P* < 0.05 was considered statistically significant.

## Results

### Den improves renal injury and fibrosis in db/db mice

**Fig. 1:**
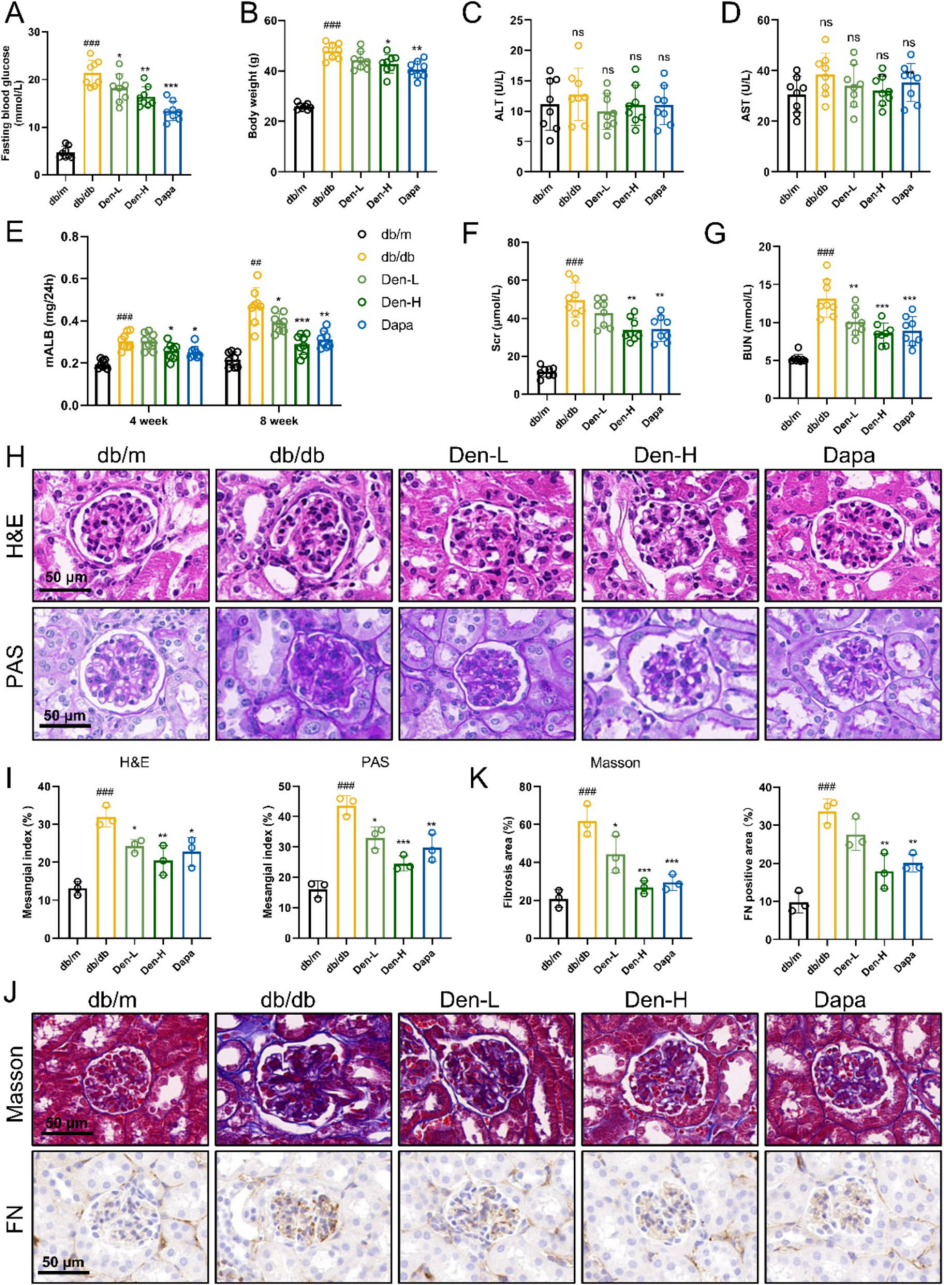
Den improves renal injury and fibrosis in db/db mice. **A** Serum glucose levels. **B** Body weight. **C**, **D** levels of ALT and AST in serum. **E** Micro-albumin levels at 4-and 8-week for drug administration. **F** Serum creatinine. **G** Blood urea nitrogen. **H, I** Representative images and quantification of mesangial index in H&E staining and PAS staining. Scale bar = 50 μm. **J**, **K** Representative images and quantification of fibrosis areas and FN-positive areas in Masson and immunohistochemistry staining. Scale bar = 50 μm. Data were expressed as Mean ± SEM, *n* = 3 or 8 for each group, ^##^*P* < 0.01, ^###^*P* < 0.001 vs. db/m group; ^*^P < 0.05, ^**^P < 0.01, ^***^P < 0.001 vs. db/db group

The therapeutic effect of Den on renal injury was evaluated in db/db mice. As illustrated in Fig. 1A, B, db/db mice exhibited significantly elevated glucose levels and body weights compared to db/m mice. However, administration with Den markedly attenuated these increases. No significant differences in ALT and AST levels were observed among db/m and db/db mice treated with or without Den (Fig. 1C, D). Biochemical analysis revealed that db/db mice had significantly increased levels of mALB, Scr, and Bun, confirming the successful establishment of an animal model for DKD. Following an 8-week treatment with Den, a significant reduction in the aforementioned biochemical markers was observed (Fig. 1E–G). Additionally, H&E and PAS staining demonstrated that Den effectively mitigated mesangial expansion, glomerular basement membrane thickening, and glycogen deposition in db/db mice (Fig. 1H, I). Furthermore, Masson staining and immunohistochemistry staining for FN revealed that Den treatment significantly reduced fibrosis area and FN-positive area, thereby inhibiting renal fibrosis (Fig. 1K–J). In summary, these findings indicate that Den exerts a significant therapeutic effect on renal injury and fibrosis in DKD.

### Den alleviates glomerular senescence in db/db mice.

**Fig. 2:**
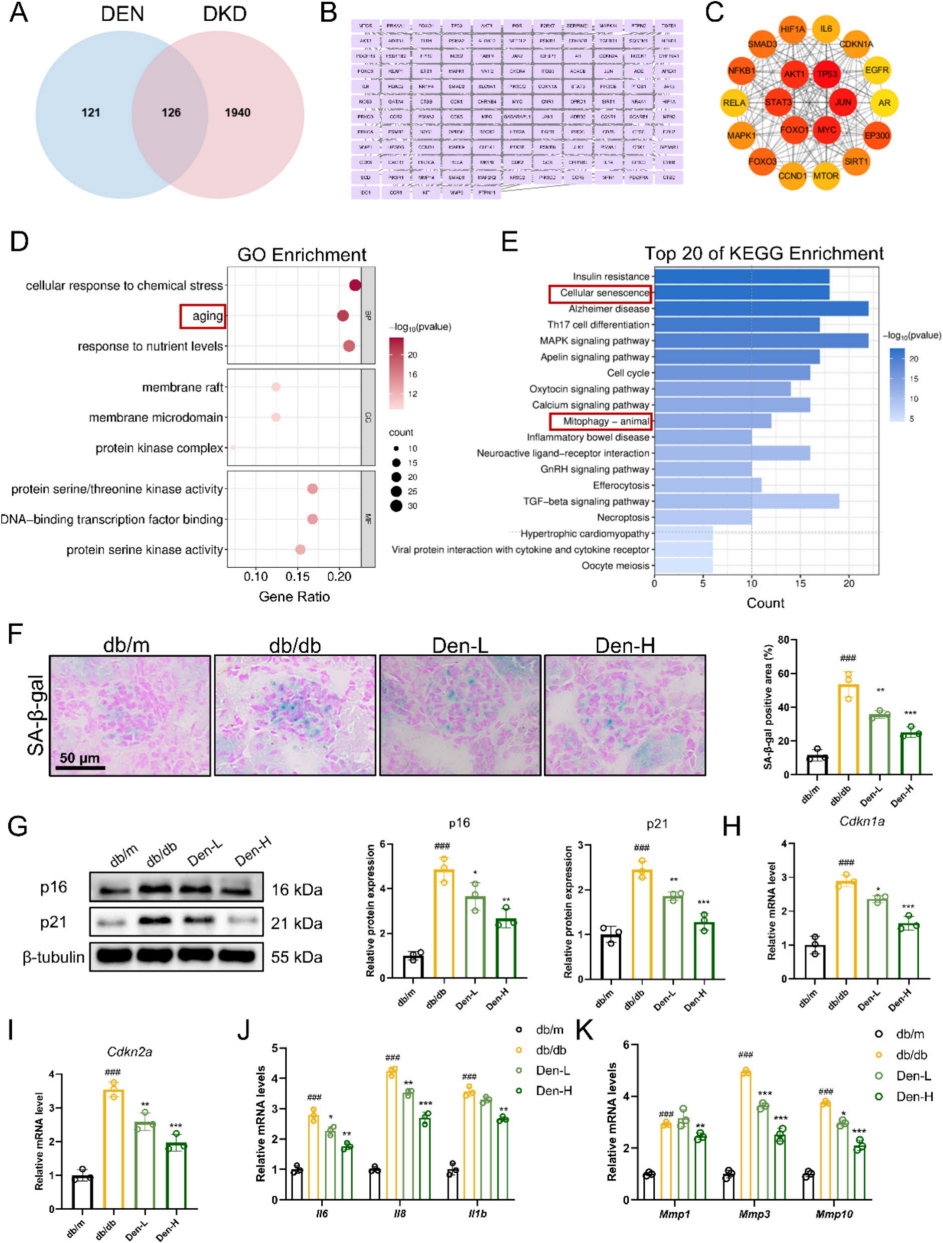
Den alleviates glomerular senescence in db/db mice. **A** Venn diagram of Den-predicted target set and DKD-predicted target set. **B** 126 intersecting predictive targets. **C** The protein–protein interaction network of the core potential targets. **D** GO enrichment analysis. **E** KEGG enrichment analysis. **F** Representative images and quantification of SA-β-gal staining. Scale bar = 50 μm. **G** Senescence-associated proteins (p16 and p21) expressions were examined by western blot and quantification. **H, I** Relative mRNA levels of *Cdkn1a* and *Cdkn2a* examined by RT-qPCR. **J, K** Relative mRNA levels of *Il6*, *Il8*, *Il1b*, *Mmp1*, *Mmp3*, and *Mmp10* examined by RT-qPCR. Data were expressed as Mean ± SEM, *n* = 3 for each group, ^###^*P* < 0.001 vs. db/m group; ^*^P < 0.05, ^**^P < 0.01, ^***^P < 0.001 vs. db/db group

Network pharmacology analysis was conducted to explore the underlying mechanisms of Den’s nephroprotective effects. A total of 126 potential targets for Den in treating DKD were predicted using Swiss Target Prediction, PharmMapper, GeneCards, and OMIM databases (Fig. 2A, B). The protein–protein interaction (PPI) network of the core potential targets is presented in Fig. 2C, including targets related to senescence (CDKN1A, TP53, CCND1) and SASP (IL6). GO and KEGG enrichment analyses revealed that Den’s nephroprotective effects may be associated with cellular senescence and mitophagy processes (Fig. 2D, E). Subsequently, SA-β-Gal staining was performed to evaluate the beneficial effect of Den on glomerular senescence, which revealed a significant decrease in the SA-β-Gal-positive areas following administration with Den (Fig. 2F). Additionally, an 8-week treatment with Den resulted in a significant reduction in both the expression of senescence-associated proteins (p16 and p21) and the mRNA levels of *Cdkn1a* and *Cdkn2a* (Fig. 2G–I). Moreover, Den also diminished the mRNA levels of inflammatory cytokines (*Il6*, *Il8*, *Il1b*), and matrix metalloproteinases (*Mmp1*, *Mmp3*, and *Mmp10*) in the cortex of db/db mice (Fig. 2J, K). Overall, our data indicate that Den effectively alleviates glomerular senescence in db/db mice.

### Den inhibits HG-induced endothelial senescence in HUVECs

**Fig. 3:**
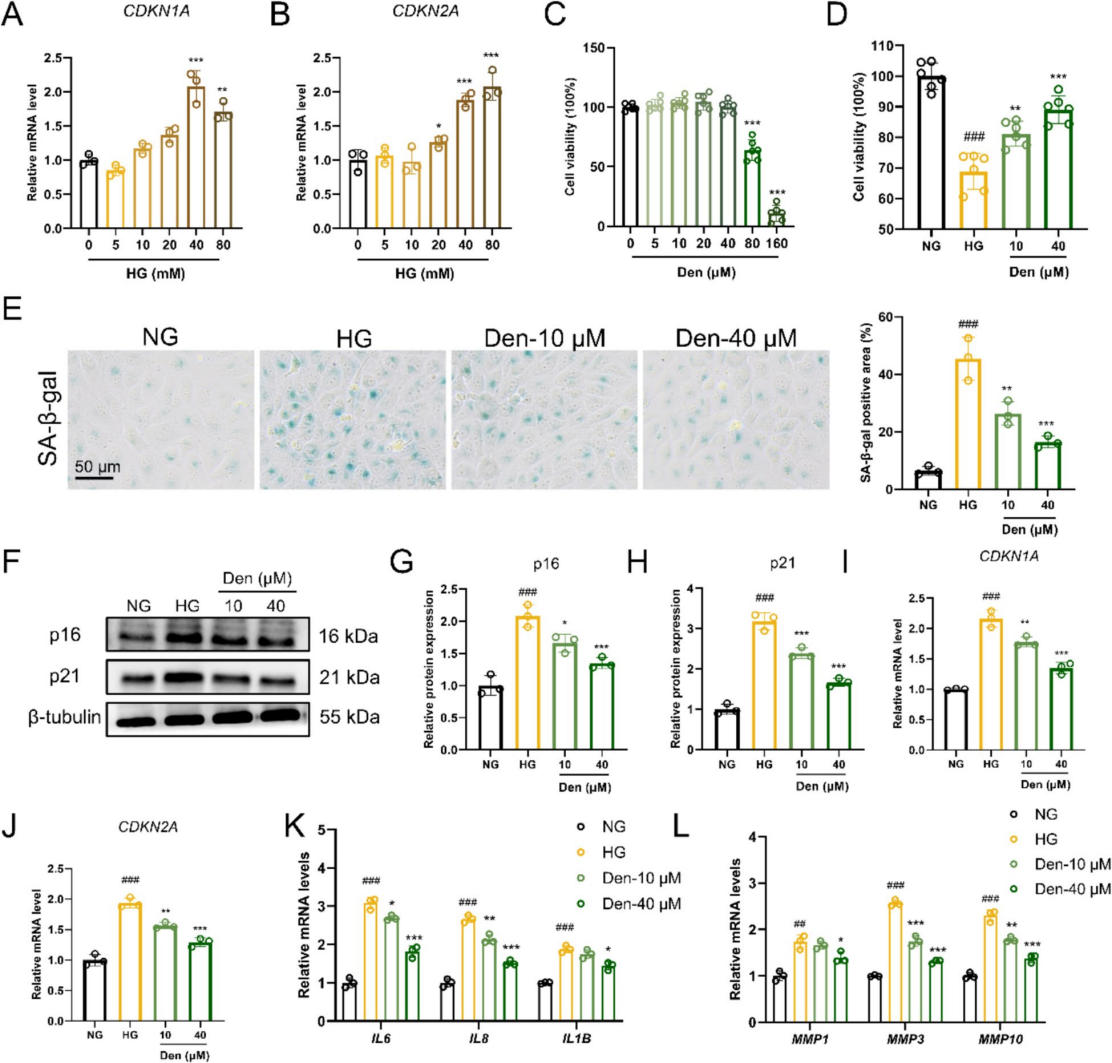
Den inhibits HG-induced endothelial senescence in HUVECs. **A, B** HUVECs were induced with or without HG (5, 10, 20, 40, and 80 mM) for 48 h. Relative mRNA levels of *CDKN1A* and *CDKN2A* examined by RT-qPCR. **C** Cell viability of HUVECs treated with Den (0, 5, 10, 20, 40 μM). **D** Cell viability of HG-induced HUVECs following Den treatment. **E** Representative images and quantification of SA-β-gal staining. Scale bar = 50 μm. **F** Senescence-associated proteins (p16 and p21) expressions were examined by western blot. **G, H** Quantification of proteins expression in F. **I, J** Relative mRNA levels of *CDKN1A* and *CDKN2A* examined by RT-qPCR. **K, L** Relative mRNA levels of *IL6*, *IL8*, *IL1B*, *MMP1*, *MMP3*, and *MMP10* examined by RT-qPCR. Data were expressed as Mean ± SEM, *n* = 3 or 6 for each group, ^##^*P* < 0.01, ^###^*P* < 0.001 vs. NG group; ^*^P < 0.05, ^**^P < 0.01, ^***^P < 0.001 vs. HG group

The HUVECs were exposed to varying concentrations of HG (5, 10, 20, 40, and 80 mmol/L) in vitro to simulate endothelial cell injury under diabetic conditions. As shown in Fig. 3A, B, HG (40 mmol/L) induced a significant increase in *CDKN1A* and *CDKN2A* mRNA levels in HUVECs, which was used for the following experiments. No statistically significant differences were observed in the cytotoxicity assay between HUVECs treated with different concentrations of Den (0, 5, 10, 20, 40 μmol/L), respectively (Fig. 3C). In Fig. 3D, Den (10 and 40 μmol/L) significantly rescued the decrease in cell viability induced by HG. Meanwhile, SA-β-Gal staining results revealed a significant decrease in the SA-β-Gal-positive areas following administration with Den (Fig. 3E). As evidenced by western blot and RT-qPCR analysis, Den exhibited a remarkable inhibition of senescence-associated proteins (p16 and p21) and genes (*CDKN1A* and *CDKN2A*) (Fig. 3F–J). Additionally, the mRNA levels of SASP (*IL6*, *IL8*, *IL1B*, *MMP1*, *MMP3*, and *MMP10*) were increased in HG-induced HUVECs; however, Den significantly reduced this expression (Fig. 3K–L). In conclusion, these results indicate that Den inhibits HG-induced endothelial senescence.

### Den rescues HG-induced endothelial senescence by improving mitophagy

**Fig.4:**
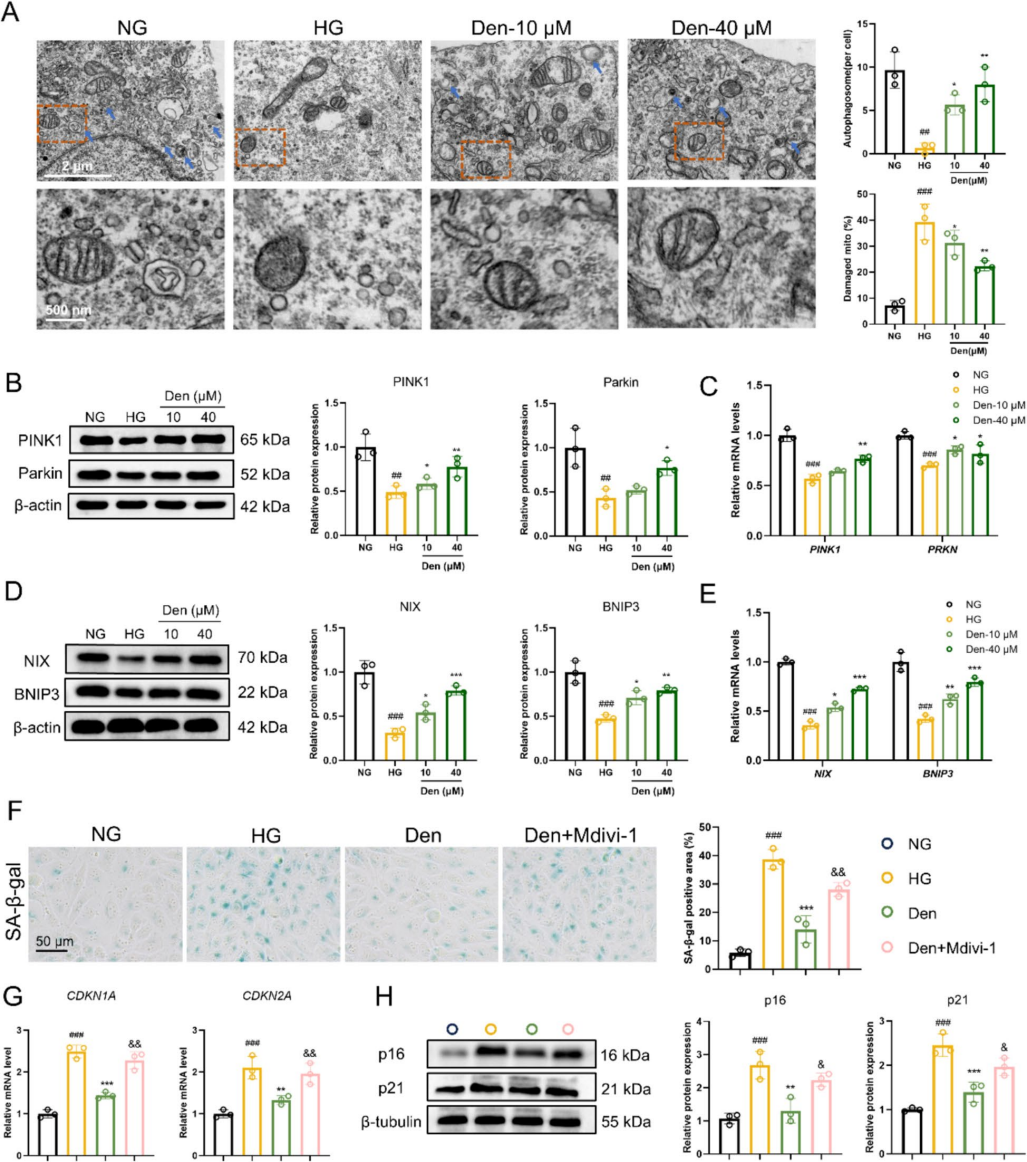
Den rescues HG-induced endothelial senescence by improving mitophagy. **A** Representative image of TEM and quantification of autophagosome and damaged mitochondria; Scale bar, 2 μm and 500 nm. **B** Mitophagy-associated proteins (PINK1 and Parkin) expressions were examined by western blot and quantification. **C** Relative mRNA levels of *PINK1* and *PRKN* examined by RT-qPCR. **D** Mitophagy-associated proteins (NIX and BNIP3) expressions were examined by western blot and quantification. **E** Relative mRNA levels of *NIX* and *BNIP3* assessed by RT-qPCR. **F** Representative images and quantification of SA-β-gal staining. Scale bar = 50 μm. **G** Relative mRNA levels of *CDKN1A* and *CDKN2A* assessed by RT-qPCR. **H** Senescence-associated proteins (p16 and p21) expressions were examined by western blot and quantification. Data were expressed as Mean ± SEM, *n* = 3 for each group, ^##^*P* < 0.01, ^###^*P* < 0.001 vs. NG group; ^*^P < 0.05, ^**^P < 0.01, ^***^P < 0.001 vs. HG group; ^&^*P* < 0.05, ^&&^*P* < 0.01 vs. Den group

Normal mitophagy maintains cellular homeostasis; however, TEM analysis indicated that the number of lysosomes containing engulfed mitochondria (indicated by blue arrows) was significantly reduced in HG-induced HUVECs compared to the control group, and this reduction was markedly restored following Den treatment. Additionally, Den treatment significantly attenuated HG-induced mitochondrial damage, as demonstrated by a decreased rate of damaged mitochondria (Fig. 4A). The PINK1/Parkin pathway has been largely implicated in mitophagosome formation of mitophagy [25]. Here, Western blot analysis revealed that Den significantly increased the protein expression of PINK1 and Parkin (Fig. 4B). Similarly, Den also upregulated the mRNA levels of *PINK1* and *PRKN* in HG-induced HUVECs (Fig. 4C). Mitophagy mediated by BNIP3 and NIX plays a critical role in regulating mitochondrial mass [26]. Significant upregulation of NIX and BNIP3 protein expression, as well as their mRNA levels, was observed after treatment with Den (Fig. 4D, E). Next, a mitophagy inhibitor (Mdivi-1, 5 μM) was employed to elucidate its association with cellular senescence. SA-β-Gal staining results revealed a significant increase in SA-β-Gal-positive areas after treatment with Den + Mdivi-1 compared to Den alone (Fig. 4F). Consistent with these findings, Mdivi-1 reversed the positive effects of Den, as evidenced by elevated *CDKN1A* and *CDKN2A* mRNA levels and increased p16 and p21 protein expression (Fig. 4G, H). Briefly, our findings demonstrate that Den rescues HG-induced endothelial senescence by enhancing mitophagy in HUVECs.

### Den activates SIRT1/FOXO3a pathway in HG-induced HUVECs

**Fig. 5:**
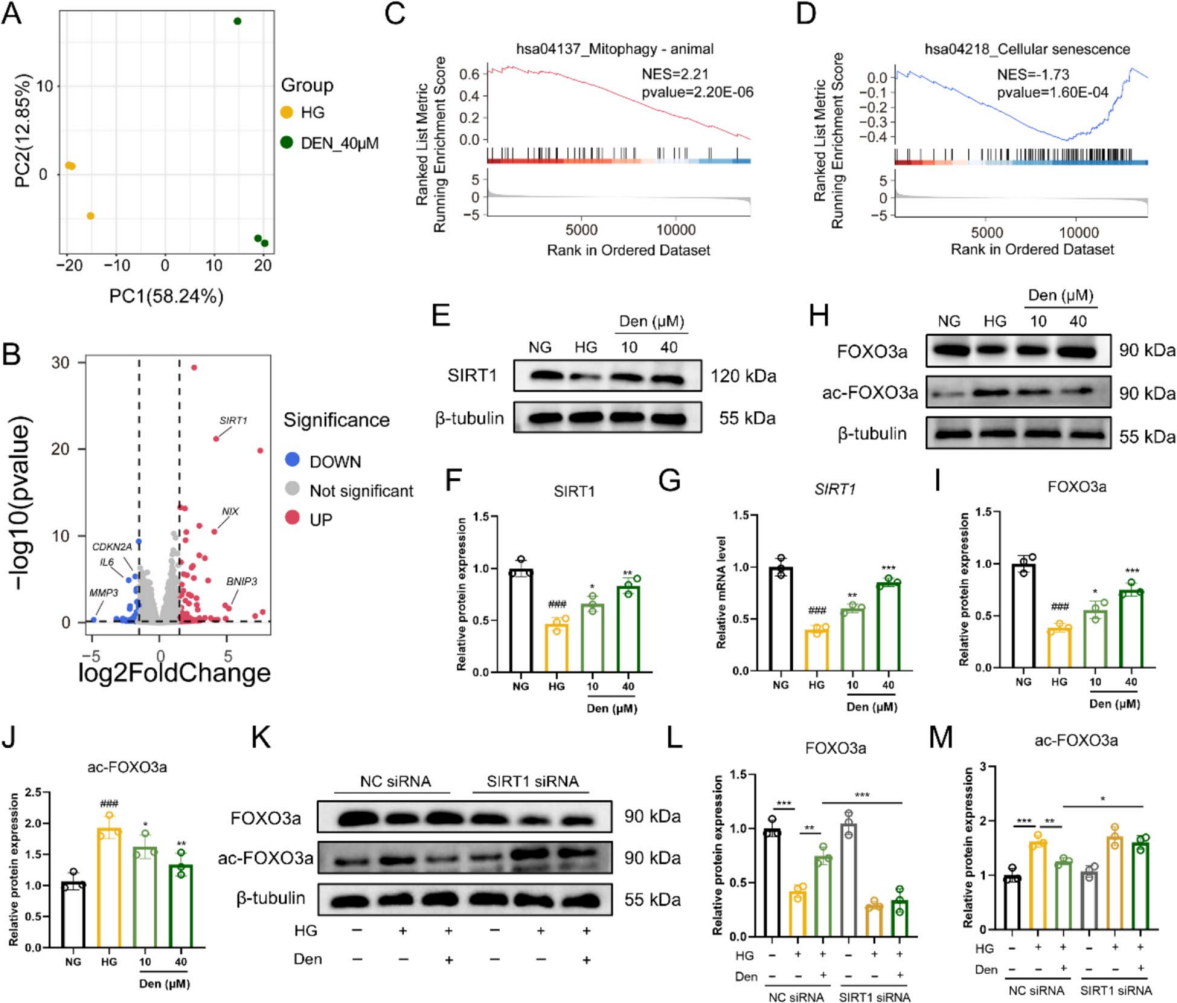
Den activates SIRT1/FOXO3a pathway in HG-induced HUVECs. **A** PCA analysis between HG group and Den group. **B** Volcano map of DEGs between HG group and Den group. **C, D** GSEA enrichment analysis of “Mitophagy-animal” pathway and “Cellular senescence” pathway. **E, F** Representative images and quantification of SIRT1 protein expression was examined by western blot. **G** Relative mRNA levels of *SIRT1* assessed by RT-qPCR. **H–J** Representative images and quantification of FOXO3a and ac-FOXO3a protein expressions were examined by western blot. **K–M** Representative images and quantification of FOXO3a and ac-FOXO3a protein expression after knockdown of SIRT1. Data were expressed as Mean ± SEM, *n* = 3 for each group, ^##^*P* < 0.01, ^###^*P* < 0.001 vs. NG group; ^*^P < 0.05, ^**^P < 0.01, ^***^P < 0.001 vs. HG group

To explore the molecular mechanisms underlying Den’s amelioration of mitophagy-mediated endothelial senescence, RNA-seq was conducted here. As shown in Fig. 5A, principal component analysis of DEGs between the HG and Den groups resulted in distinct separation of clusters without any overlap. After Den treatment, the mRNA levels of *SIRT1*, *NIX*, and *BNIP3* were significantly upregulated in HG-induced HUVECs, while the mRNA levels of *MMP3*, *IL6*, and *CDKN2A* were significantly downregulated (Fig. 5B). GSEA enrichment analysis suggested the “Mitophagy-animal” pathway was significantly upregulated, whereas the “Cellular senescence” pathway was significantly downregulated after Den treatment (Fig. 5C, D). Subsequently, the expression of SIRT1 in HG-induced HUVECs treated with or without Den was assessed, revealing a significant increase in SIRT1 at both mRNA and protein levels (Fig. 5E–G). Recent studies have reported that SIRT1 activates FOXO3a transcription by modulating its acetylation status. As shown in Fig. 5H–J, Den inhibited total FOXO3a expression while improving the ac-FOXO3a expression. Next, knockdown of SIRT1 abolished the Den-induced increase in FOXO3a expression and also reduced ac-FOXO3a protein expression (Fig. 5K–M). In summary, the above data indicate that Den activates the SIRT1/FOXO3a pathway in HG-induced HUVECs.

### Den improves mitophagy through activating the SIRT1/FOXO3a pathway

**Fig. 6:**
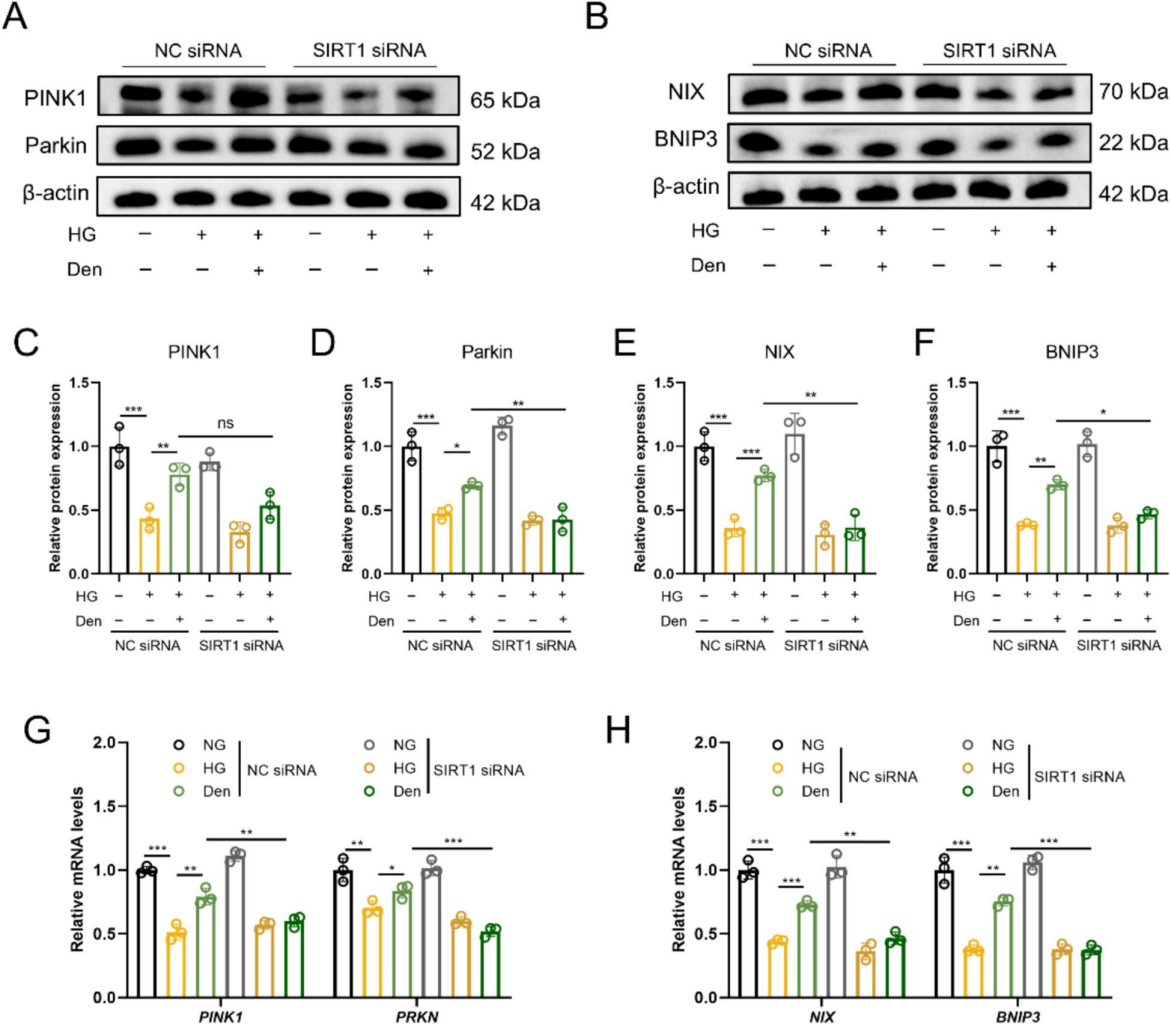
Den improves mitophagy through activating SIRT1/FOXO3a pathway. **A, B** Mitophagy-associated proteins (PINK1, Parkin, NIX, and BNIP3) expressions were examined by western blot after knockdown of SIRT1. **C–F** Quantification of proteins expression in A and B. **G, H** Relative mRNA levels of *PINK1, PRKN, NIX* and *BNIP3* assessed by RT-qPCR. Data were expressed as Mean ± SEM, *n* = 3 for each group, ^*^P < 0.05, ^**^P < 0.01, ^***^P < 0.001

To further ensure the effect of the SIRT1/FOXO3a pathway involved in Den’s positive impact on enhancing mitophagy, siRNA was used in this study to knock down the expression of SIRT1. Mitophagy-associated proteins and genes were evaluated by western blot and RT-qPCR analysis. As shown in Fig. 6A–F, Den-induced elevation of PINK1, Parkin, NIX, and BNIP3 protein expression was significantly inhibited following knockdown of SIRT1. Additionally, RT-qPCR results indicated that knockdown of SIRT1 decreased the mRNA levels of *PINK1*, *PRKN*, *NIX*, and *BNIP3*, which were upregulated by Den (Fig. 6G–H). In short, our findings demonstrate that Den improves mitophagy through the SIRT1/FOXO3a pathway.

### Den inhibits mitophagy-induced senescence by the SIRT1/FOXO3a pathway

**Fig. 7:**
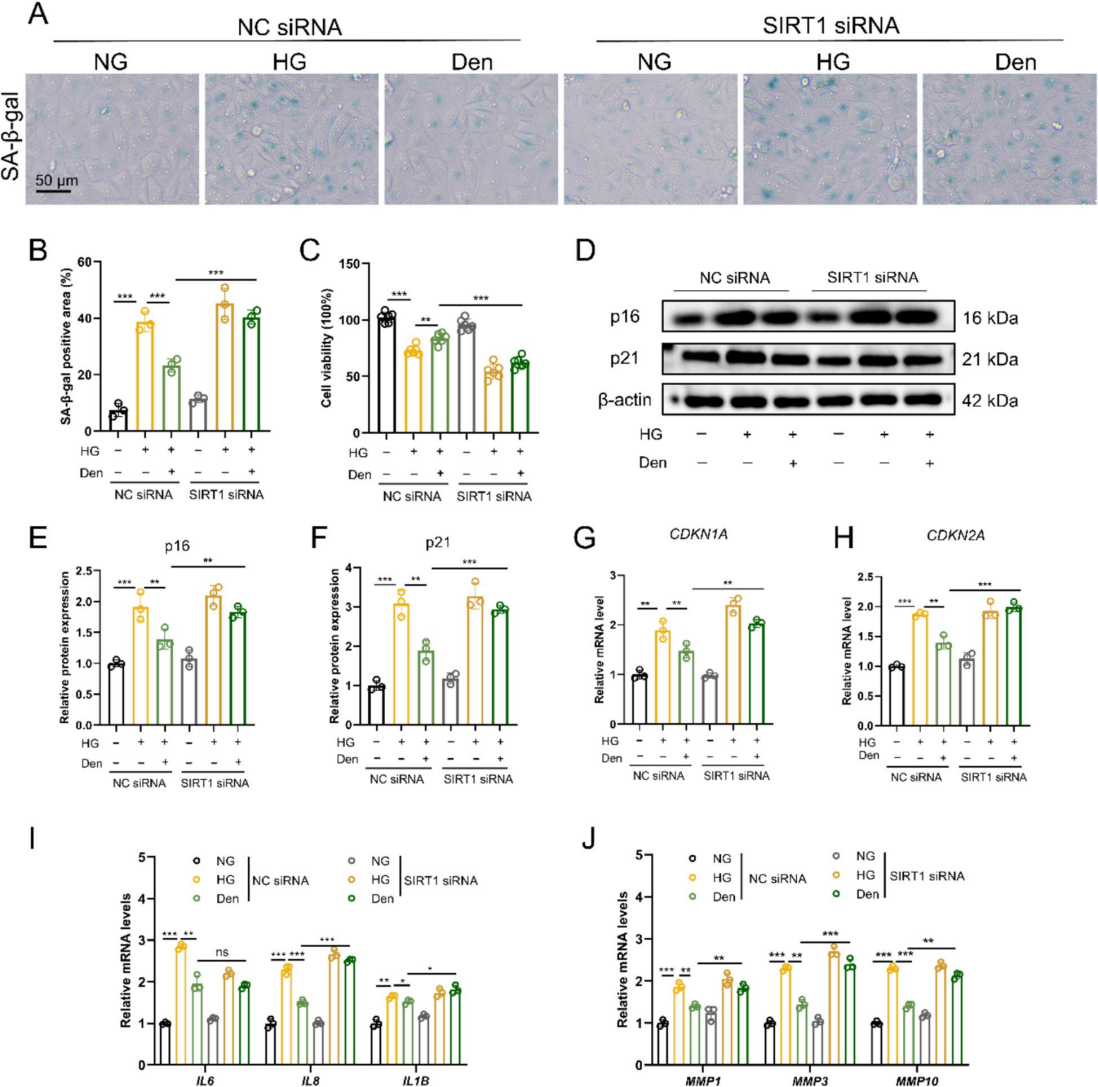
Den inhibits mitophagy-induced senescence by SIRT1/FOXO3a pathway. **A, B** Representative images and quantification of SA-β-gal staining. Scale bar = 50 μm. **C** Cell viability. **D** The protein expression of p16 and p21 were examined by western blot. **E, F** Quantification of proteins expression in (**D**). **G, H** Relative mRNA levels of *CDKN1A* and *CDKN2A* assessed by RT-qPCR. **I, J** Relative mRNA levels of *IL6*, *IL8*, *IL1B*, *MMP1*, *MMP3*, and *MMP10* assessed by RT-qPCR. Data were expressed as Mean ± SEM, *n* = 3 for each group, ^*^P < 0.05, ^**^P < 0.01, ^***^P < 0.001

After knockdown of SIRT1, SA-β-Gal staining showed a significant increase in SA-β-Gal positive area and a remarkable decrease in cell viability in HG-induced HUVECs treated with Den (Fig. 7A–C). Western blot analysis indicated that knockdown of SIRT1 improved the protein expression of p16 and p21, which were downregulated by Den. Similarly (Fig. 7D–F), the mRNA levels of CDKN1A and CDKN2A were also increased (Fig. 7G–H). Furthermore, the positive effects of Den on inhibiting SASP-associated gene (IL6, IL8, IL1B, MMP1, MMP3, and MMP10) levels were reversed after knockdown of SIRT1 (Fig. 7I, J). Overall, these data suggest that Den inhibits mitophagy-induced senescence by the SIRT1/FOXO3a pathway.

### Den ameliorates endothelial senescence in DKD mice through activating the SIRT1/FOXO3a pathway

**Fig. 8:**
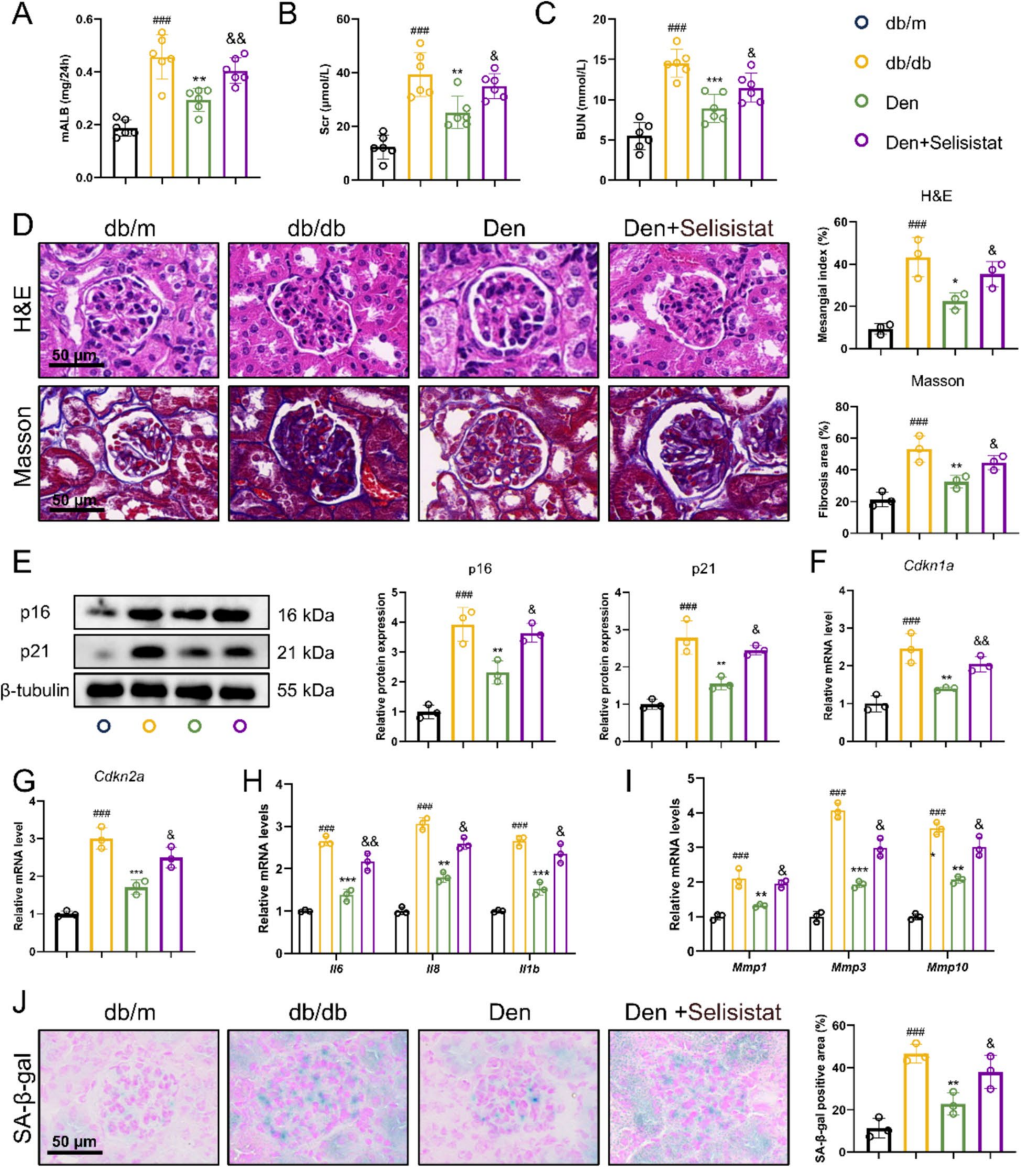
Den ameliorates endothelial senescence in DKD mice through activating SIRT1/FOXO3a pathway. **A** Micro-albumin levels. **B** Serum creatinine. **C** Blood urea nitrogen. **D** Representative images and quantification of mesangial index in H&E staining and fibrosis area in Masson staining. Scale bar = 50 μm. **E** Senescence-associated proteins (p16 and p21) expressions were examined by western blot and quantification. **F, G** Relative mRNA levels of *CDKN1A* and *CDKN2A* assessed by RT-qPCR. **H, I** Relative mRNA levels of *IL6*, *IL8*, *IL1B*, *MMP1*, *MMP3*, and *MMP10* assessed by RT-qPCR. **J** Representative images and quantification of SA-β-gal staining. Scale bar = 50 μm. Data were expressed as Mean ± SEM, *n* = 3 or 6 for each group. ^###^*P* < 0.001 vs. NG group; ^*^P < 0.05, ^**^P < 0.01, ^***^P < 0.001 vs. HG group; ^&^*P* < 0.05, ^&&^*P* < 0.01 vs. Den group

**Fig. 9:**
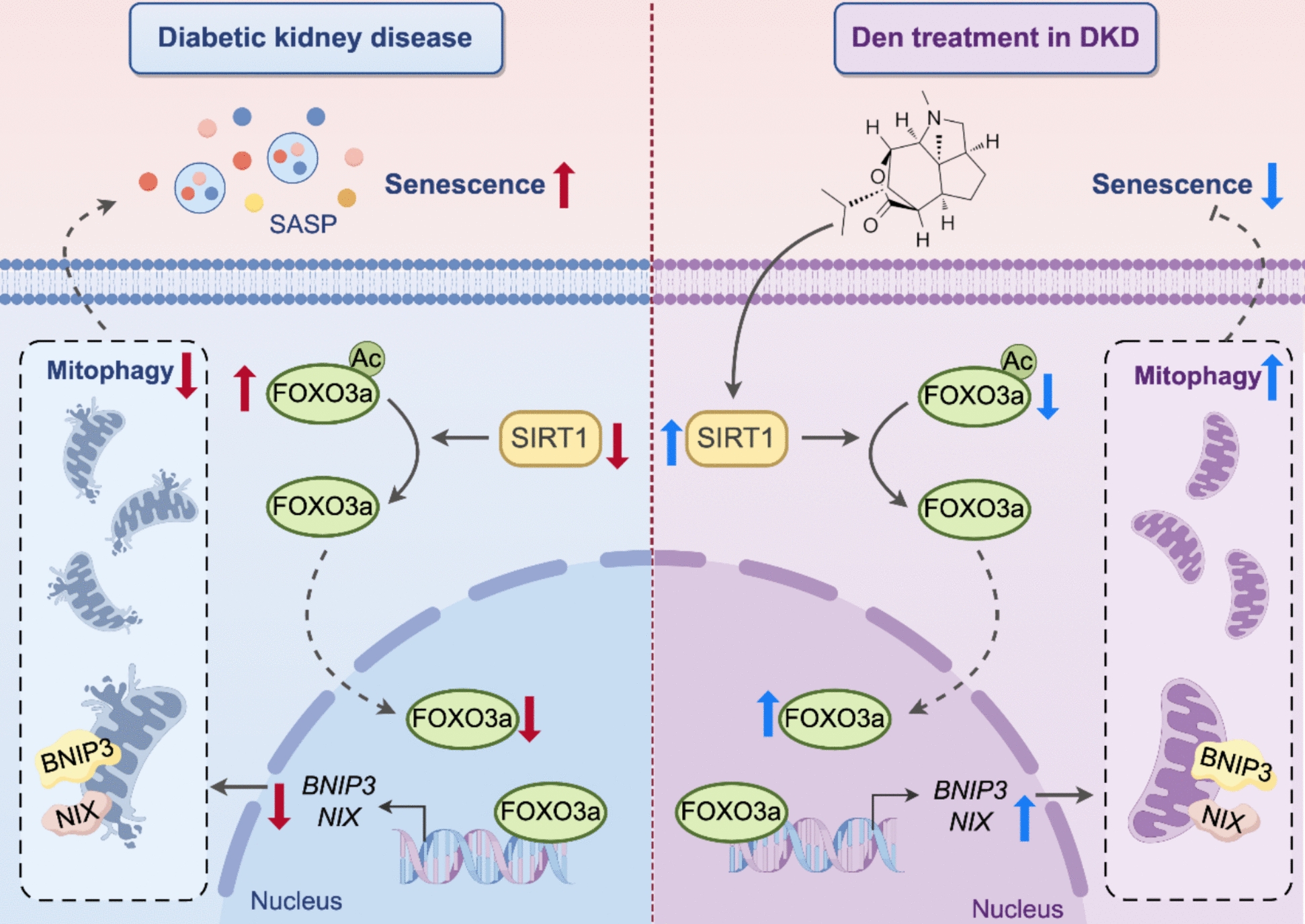
Dendrobine ameliorates mitophagy-mediated endothelial senescence in diabetic kidney disease through activating SIRT1/FOXO3a pathway

To further confirm the role of the SIRT1/FOXO3a pathway in Den-mediated inhibition of endothelial senescence, the pharmacological inhibitor of SIRT1, selisistat, was used here. As shown in Fig. 8A–C, selisistat significantly reversed the Den-induced reductions in mALB, Scr, and BUN levels. H&E and Masson staining results indicated a remarkable increase in mesangial index and fibrosis area following treatment with Den + selisistat compared to Den alone (Fig. 8D). Additionally, Western blot analysis revealed that selisistat reversed Den’s inhibitory effects on p16 and p21 protein expression, as well as *Cdkn1a* and *Cdkn2a* mRNA levels (Fig. 8E–G). Similarly, SPAP levels and SA-β-gal-positive areas exhibited consistent trends (Fig. 8H–J). In conclusion, our findings suggest that Den ameliorates endothelial senescence in DKD mice through activating the SIRT1/FOXO3a pathway (Fig. 9).

## Discussion

In this study, our findings demonstrated that Den significantly ameliorates renal injury through inhibiting endothelial senescence in DKD mice. Mechanistically, we clarified that Den ameliorated mitophagy-mediated endothelial senescence through activating the SIRT1/FOXO3a pathway. These data indicated that amelioration of endothelial senescence represented a promising potential therapeutic strategy and highlighted the potential effect of Den in inhibiting glomerular senescence in DKD.

Growing evidence has demonstrated that ameliorating renal senescence can delay the pathologic progression of DKD [12, 27]. However, previous research has primarily focused on renal tubular senescence while neglecting the role of glomerular senescence. This may be attributed to the fact that, compared to the complex structure of the glomerulus, the renal tubule consists only of epithelial cells, making it a clearer object for study [28]. Additionally, renal tubules may be more susceptible to damage from glucotoxicity and senescence, as indicated by the clinical findings showing altered urinary composition in diabetic patients, particularly with markers of tubular injury such as beta-2 microglobulin appearing before obvious glomerular pathologic changes [29]. Nevertheless, these results highlighted the importance of tubular senescence, while we cannot deny the involvement of glomerular senescence in DKD development. In this study, our results demonstrated that Den significantly inhibited glomerular senescence after improving renal function in DKD mice. Similarly, another research reported that M1 macrophages accelerated renal glomerular endothelial senescence through reactive oxygen species accumulation in streptozotocin-induced diabetic mice [30]. These results highlighted the contributory effect of glomerular endothelial senescence in DKD development and underscore the feasibility of targeting this process for disease treatment.

SIRT1 represents a promising therapeutic target for the development of drugs against DKD. Several studies on single nucleotide polymorphisms have revealed an association between SIRT1 and DKD. The results demonstrated that SIRT1 gene variant rs10823108 and FoxO1 gene variant rs17446614 may be associated with DKD [30], while SIRT1 polymorphism is related to progression of albumin-creatinine ratio [31]. Animal experiments showed that SIRT1 (endo^−/−^) mice exhibited significant acute renal functional deterioration followed by an exaggerated fibrotic response compared to control animals [32, 33]. However, activation of SIRT1 signaling by calcium dobesilate effectively inhibits renal fibrosis and delays peritubular capillary loss in the kidneys [34]. Additionally, glomerular expression of SIRT1 is reduced in human diabetic glomeruli, and podocyte-specific loss of SIRT1 aggravated albuminuria and kidney disease progression in diabetic mice. Both podocyte-specific overexpression of SIRT1 and BT175 treatment attenuated diabetes-induced podocyte loss and reduced oxidative stress in OVE26 mouse glomeruli [35]. In our study, we observed that Den ameliorated mitophagy-mediated endothelial senescence through activating the SIRT1/FOXO3a pathway. Briefly, these findings suggest that targeting SIRT1 is a potential therapeutic approach for treating DKD.

It is worth mentioning that several limitations still existed here. Another potential mechanism underlying the promotion of mitophagy by Den may is the inhibition of FOXO3a phosphorylation. Previous research has shown that the phosphorylation of FOXO3a at Thr32, Ser253, and Ser315, primarily mediated by Akt/PKB kinase, facilitates its translocation from the nucleus to the cytoplasm, thereby suppressing its transcriptional activity [36]. Notably, Den has been reported to inhibit the phosphorylation of Sch9, a protein functionally related to FOXO3a [37]. Therefore, the molecular mechanisms underlying Den’s anti-aging effects warrant further investigation. Additionally, the long-term safety profile of Den remains to be fully elucidated. Our findings indicate that no significant toxic side effects were observed in mice following eight weeks of oral administration; however, long-term toxicity studies are still required in future research.

Taken together, our results firstly demonstrate that inhibition of glomerular senescence effectively improves DKD and elucidate the molecular mechanism by which Den ameliorates endothelial senescence through activating the SIRT1/FOXO3a pathway. These findings provide innovative insights into therapeutic strategies for DKD and underscore Den as a potential therapeutic pharmacological intervention in DKD.

## Data Availability

The datasets used and analyzed during the current study are available from the corresponding author on reasonable request. No datasets were generated or analysed during the current study.

## Abbreviations

DKD: Diabetic kidney disease
Den: Dendrobine
SA-β-Gal: Senescence-associated β-galactosidase
TEM: Transmission electron microscopy
eGFR: Estimated glomerular filtration rate
CKD: Chronic kidney disease
SASP: Senescence-associated secretory phenotype
ROS: Reactive oxygen species
TCM: Traditional Chinese medicine
H&E: Hematoxylin-eosin
PAS: Periodic Acid-Schiff
HUVECs: Human umbilical vein endothelial cells
HG: High-glucose
NG: No-glucose
PCA: Principal component analysis
DEGs: Differentially expressed genes
GO: Gene Ontology
GSEA: Gene set enrichment analysis
